# Resveratrol and Its Nitric Oxide–Donor Hybrid as an Emerging Therapy for Oxidative-Stress-Driven Priapism in Sickle Cell Disease

**DOI:** 10.3390/antiox14101213

**Published:** 2025-10-08

**Authors:** Carolina Oliveira Splendore, Mariana G. de Oliveira, Fernando Ferreira Costa, Fábio Henrique Silva

**Affiliations:** 1 Laboratory of Pharmacology, São Francisco University Medical School, Bragança Paulista 12916-900, SP, Brazil; 2 Hematology and Hemotherapy Center, University of Campinas, Campinas 13083-878, SP, Brazil

**Keywords:** 3-nitrotyrosine, cGMP, hemoglobin, reactive oxygen species, corpus cavernosum

## Abstract

Priapism is a frequent and debilitating complication in patients with sickle cell disease (SCD), characterized by recurrent ischemic episodes that can culminate in fibrosis of the erectile tissue and irreversible erectile dysfunction. Despite significant advancements in the management of acute episodes, current therapies remain largely ineffective in preventing recurrences, emphasizing the need for novel strategies that target the underlying pathophysiology. This narrative review describes the mechanistic links between oxidative stress and nitric oxide (NO) dysregulation in the pathogenesis of SCD-associated priapism, with a particular focus on the NO–cyclic guanosine monophosphate (cGMP)–phosphodiesterase type 5 (PDE5) signaling axis. We analyze preclinical evidence supporting resveratrol, a natural polyphenolic compound, as well as its NO-donor hybrid derivatives, as emerging therapeutic candidates. Additionally, we discuss the potential of combining resveratrol with current treatment approaches, and address the translational challenges that must be overcome to move from preclinical data to clinical application. Taken together, the evidence presented in this review supports resveratrol-based therapies as a promising approach for oxidative-stress-driven priapism in SCD and delineates critical perspectives for their further investigation.

## 1. Introduction

Sickle cell disease (SCD) is the most common hereditary hemoglobinopathy, affecting millions of individuals worldwide. Caused by a single point mutation in the β-globin gene, SCD results in the production of hemoglobin S (HbS), an abnormal variant that polymerizes under deoxygenated conditions. This intracellular polymerization increases red blood cell (RBC) rigidity and fragility, distorting the characteristic biconcave morphology into sickle-shaped forms. These pathologically altered erythrocytes exhibit reduced deformability, promoting chronic hemolysis, endothelial dysfunction, microvascular occlusion, and cumulative organ damage, hallmarks that contribute to substantial morbidity and premature mortality in affected individuals [1].

Ischemic priapism is a well-recognized complication of SCD, often associated with significant physical and psychological morbidity. Characterized by a persistent penile erection unrelated to sexual arousal, ischemic priapism affects up to 48% of male patients with SCD, particularly during adolescence and early adulthood [2,3]. The ischemic form, which is the most common in this context, results from impaired venous outflow and blood stasis within the corpora cavernosa, leading to prolonged and often painful episodes. Recurrent episodes known as stuttering priapism consist of frequent, painful erections lasting less than four hours and may lead to fibrosis of the erectile tissue and irreversible erectile dysfunction, severely compromising reproductive health and quality of life [3]. Although acute episodes can be managed with self-administered intracavernosal injections of α-adrenergic sympathomimetics, such as phenylephrine, this pharmacological intervention does not constitute a true preventive strategy, as it does not address the underlying pathophysiology of SCD-associated priapism [3,4].

Historically, priapism in SCD was largely attributed to mechanical venous occlusion driven by interactions among sickled erythrocytes, endothelial cells, leukocytes, and platelets. However, recent advances have revealed a more nuanced pathophysiology, implicating dysregulation of the molecular signaling pathways that govern penile smooth muscle tone [5]. Central to this dysfunction is the aberrant over-relaxation of the corpus cavernosum smooth muscle, mediated by disturbances in nitric oxide (NO) signaling and exacerbated by oxidative stress [6].

Oxidative stress plays a pivotal role in the pathophysiology of priapism in SCD, marked by excessive production of reactive oxygen species (ROS) and diminished antioxidant defenses. This redox imbalance aggravates hemolysis-induced vascular injury and reduces endothelial NO bioavailability in the corpus cavernosum [7,8,9,10]. The resulting impairment of NO–cyclic guanosine monophosphate (cGMP) signaling is a key driver of priapism in SCD [5]. Preclinical evidence demonstrates that strategies aimed at restoring redox homeostasis, enhancing endothelial NO signaling, and normalizing phosphodiesterase type 5 (PDE5) expression effectively attenuate the priapism phenotype, highlighting oxidative stress as a therapeutic target [9,10,11,12,13].

In this context, resveratrol (3,5,4′-trihydroxystilbene), a polyphenol present in grapes, berries, and peanuts, has emerged as a promising therapeutic candidate. By enhancing endothelial nitric oxide synthase (eNOS) activity, reducing oxidative stress, and improving NO bioavailability, resveratrol directly addresses the molecular drivers of priapism in SCD [14,15,16,17,18]. Recent preclinical findings demonstrated that resveratrol treatment attenuated the priapism phenotype in transgenic SCD mice by restoring NO–cGMP signaling and downregulating NADPH oxidase 2 expression [19]. Furthermore, hybrid compounds that combine resveratrol with nitric oxide–donor moieties have been developed to amplify these effects [20,21]. This review explores the mechanistic links between oxidative stress and priapism in SCD and highlights resveratrol and its NO-donor hybrids as emerging therapeutic strategies.

## 2. Molecular Mechanisms of Penile Erection

**Figure 1:**
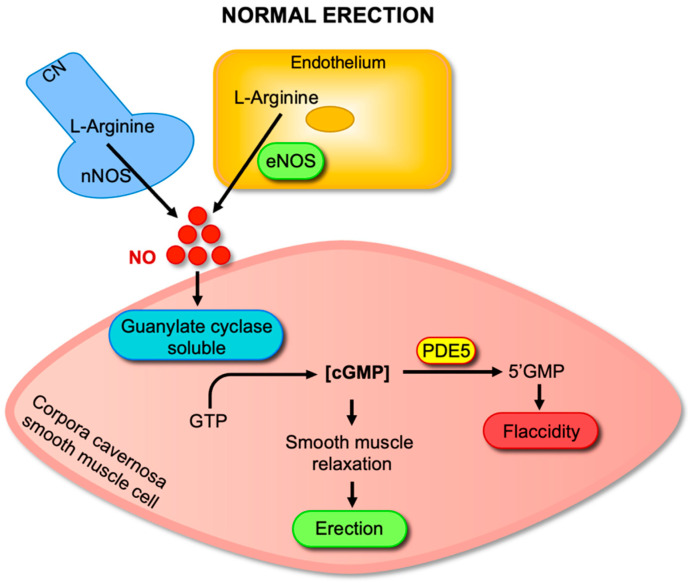
NO–cGMP–PDE5 signaling pathway in penile erection. Sexual stimulation activates neuronal nitric oxide synthase (nNOS) in cavernosal nerves and endothelial nitric oxide synthase (eNOS) in endothelial cells, leading to the conversion of L-arginine into nitric oxide (NO). NO diffuses into cavernosal smooth muscle cells and stimulates soluble guanylate cyclase (sGC), which catalyzes the conversion of guanosine-5′-triphosphate (GTP) into cyclic guanosine monophosphate (cGMP). Increased cGMP levels activate downstream targets, resulting in corpus cavernosum smooth muscle relaxation, arterial dilation, and penile erection. Phosphodiesterase type 5 (PDE5) terminates this signal by degrading cGMP into 5′-GMP, thereby returning the penis to flaccidity.

A penile erection results from a complex interplay of vascular, neural, and hormonal factors [22]. Among the several pathways involved, the NO-cGMP-PDE5 signaling cascade represents the principal molecular axis mediating penile erection (Figure 1). Two critical NO synthase (NOS) enzymes exist in the penis: endothelial (eNOS) and neuronal (nNOS). These enzymes are pivotal in starting and sustaining an erection, catalyzing the transformation of L-arginine into L-citrulline and NO [23]. Upon sexual stimulation, NO diffuses freely across cell membranes and activates sGC in adjacent cavernosal smooth muscle cells. Activated sGC catalyzes the conversion of guanosine-5′-triphosphate (GTP) into cGMP, leading to a rapid increase in intracellular cGMP levels. cGMP is as a second messenger that acitivates cGMP-dependent protein kinase (PKG), which influences various proteins that mediate muscle relaxation, such as myosin light chain phosphatase and potassium channels [22]. Collectively, these molecular events culminate in cavernosal smooth muscle relaxation, arterial dilation, and sinusoidal blood filling, which together generate penile rigidity. The duration and magnitude of the erectile response are regulated by PDE5, a cGMP-specific phosphodiesterase abundantly expressed in cavernosal smooth muscle. PDE5 hydrolyzes cGMP into its inactive form, 5′-GMP, thereby terminating the NO-mediated signal and penile erection. This finely tuned balance between cGMP synthesis by sGC and degradation by PDE5 ensures the transient nature of penile erection [23].

## 3. Pathophysiology of SCD-Associated Priapism: Role of Oxidative Stress and Nitric Oxide Dysregulation

**Figure 2:**
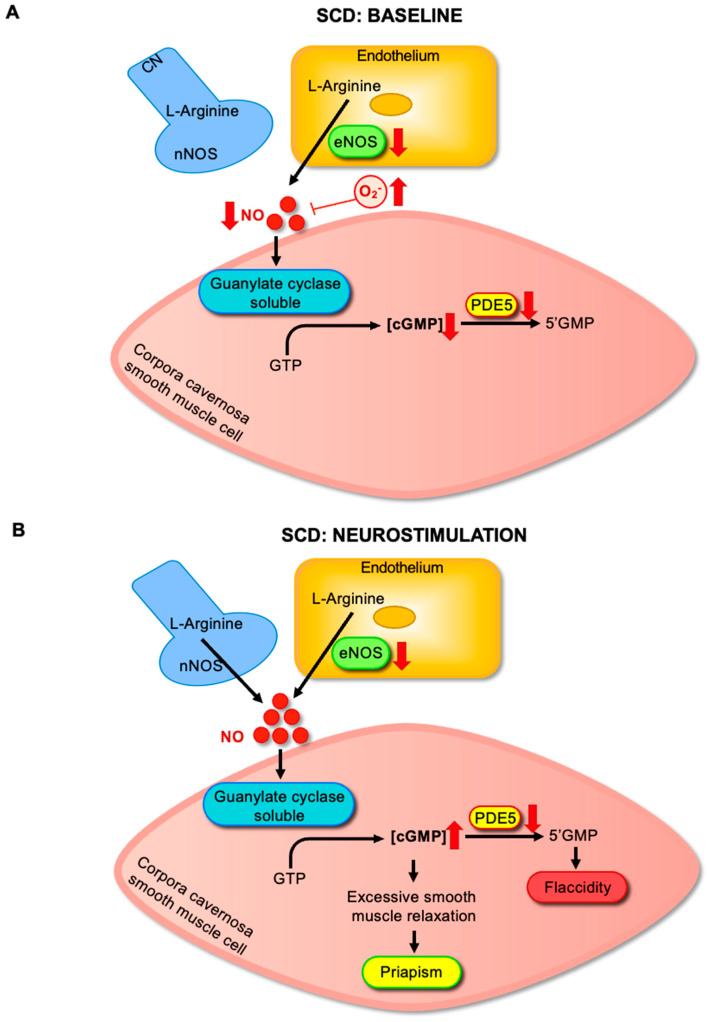
(**A**) Altered NO–cGMP–PDE5 signaling in sickle cell disease (SCD) at baseline. In SCD, reduced endothelial nitric oxide synthase (eNOS) activity and increased oxidative stress, driven primarily by elevated superoxide (O_2_^−^) levels, and diminished NO bioavailability. Lower NO levels impair soluble guanylate cyclase (sGC) activation and limit cGMP production in cavernosal smooth muscle cells. Reduced cGMP levels subsequently lead to the downregulation of PDE5 expression and activity. (**B**) Dysregulated NO–cGMP–PDE5 signaling during neurostimulation in sickle cell disease (SCD). Upon neurostimulation, neuronal nitric oxide synthase (nNOS) and endothelial nitric oxide synthase (eNOS) generate NO, which activates soluble guanylate cyclase (sGC) and increases cGMP production in cavernosal smooth muscle cells. However, in SCD, the downregulation of PDE5 impairs cGMP degradation, leading to its excessive accumulation. This imbalance drives sustained relaxation of cavernosal smooth muscle, predisposing individuals to prolonged erections and the development of priapism.

Experimental and clinical evidence has established that priapism in SCD is primarily driven by dysregulation of the NO–cGMP–PDE5 signaling axis [6,24,25]. Reduced NO bioavailability in the corpus cavernosum of SCD patients and animal models leads to chronically diminished basal cGMP levels [9,11,12,13,24]. Because PDE5 expression is positively regulated by intracellular cGMP concentrations, reduced basal levels result in marked downregulation of PDE5 protein in cavernous smooth muscle cells [26] (Figure 2A). As a result, upon sexual or nocturnal stimuli that transiently increase NO, cGMP accumulates abnormally, triggering exaggerated smooth muscle relaxation and initiating prolonged, unregulated penile erection [5] (Figure 2B).

These pathophysiological features have been extensively characterized in transgenic SCD mouse models, including both the Berkeley and Townes strains, which exhibit reduced PDE5 expression and heightened erectile responses [9,24,27]. Functional ex vivo studies have demonstrated augmented nitrergic relaxation following electrical field stimulation, as well as enhanced endothelium-dependent (acetylcholine-induced) and endothelium-independent (NO donor-mediated) relaxation of corpus cavernosum strips [9,10,11,13]. Consistently, in vivo assessments demonstrate elevated intracavernosal pressure (ICP) in response to cavernous nerve stimulation in SCD mice, corroborating the hyperresponsiveness of erectile tissue in this context [11,13,28].

A key pathophysiological determinant of priapism in the penile tissue of individuals with SCD is reduced NO bioavailability due to the accumulation of ROS [6]. Multiple mechanisms contribute to oxidative stress in SCD penile tissue. A major factor is the chronic intravascular hemolysis characteristic of SCD, which leads to elevated levels of cell-free hemoglobin in the plasma [29]. This hemoglobin binds and inactivates NO, thus reducing NO bioavailability [10,30]. In parallel, enzymatic sources of ROS are upregulated, particularly xanthine oxidase and NADPH oxidase [8,9,19]. Xanthine oxidase catalyzes the sequential oxidation of hypoxanthine and xanthine to uric acid, generating superoxide anion as a byproduct [31]. This superoxide rapidly reacts with NO to form peroxynitrite, a highly reactive nitrogen species that further depletes NO and promotes oxidative injury in penile tissues [32].

Among enzymatic sources, NADPH oxidase significantly contributes to priapism through its superoxide production in SCD. In particular, the NOX2 isoform, which requires the assembly of membrane-bound subunits (gp91^phox^ and p22^phox^) and cytosolic subunits (p47^phox^, p67^phox^, p40^phox^, Rac1/Rac2). Increased expression of gp91^phox^, p47^phox^, and p67^phox^ has been consistently reported in penile tissue from both SCD patients and mouse models, contributing to sustained superoxide production [8,10,11,13,25,33].

Another important contributor to oxidative stress in the penile tissue of individuals with SCD is uncoupled eNOS [8]. Under physiological conditions, eNOS generates NO to maintain vascular homeostasis. However, in the oxidative environment characteristic of SCD, eNOS becomes uncoupled and begins producing superoxide instead of NO, further amplifying ROS levels [5,8]. This shift exacerbates formation of peroxynitrite, which triggering lipid peroxidation and protein nitration, which together compromise membrane integrity and disrupt intracellular signaling pathways [32]. Elevated levels of 3-nitrotyrosine (3-NT), a stable end-product of peroxynitrite-mediated protein modification, have been consistently detected in the corpus cavernosum of SCD mice, serving as a biomarker of nitrosative stress and molecular dysfunction [10,11,19,25].

Building upon these established mechanisms, further molecular alterations have been identified that contribute to priapism in SCD. Opiorphin, an endogenous pentapeptide that inhibits neutral endopeptidase activity, is upregulated in SCD and has been implicated in the prolongation of smooth muscle relaxation through enhanced pro-erectile signaling [34]. Adenosine is markedly elevated in the penile tissue of SCD mice and contributes to priapism by promoting smooth muscle relaxation via A_2_B receptor-mediated cAMP signaling [35]. Impairments in the RhoA/Rho-kinase pathway, which normally facilitates detumescence through contractile signaling, have also been observed in SCD mice, shifting the balance toward persistent smooth muscle relaxation [36]. Moreover, decreased expression of tyrosine hydroxylase, the rate-limiting enzyme in catecholamine biosynthesis, indicates compromised sympathetic neurotransmission, further impairing detumescence mechanisms [4].

Collectively, these findings illustrate the complex and interrelated mechanisms underlying priapism in SCD. The combined effects of oxidative stress, reduced NO signaling, impaired contractile pathways, and dysregulated neuromodulatory control create a pathological state that favors recurrent, unregulated penile erections. These mechanistic insights highlight the urgent need for therapeutic approaches that target not only NO-cGMP-PDE5 dysregulation but also the broader redox and neuromodulatory landscape of SCD-associated priapism.

## 4. Pharmacological Profile of Resveratrol

Resveratrol is a naturally occurring polyphenolic stilbene predominantly found in grapes, berries, peanuts, and several medicinal plants. It has been extensively studied due to its broad spectrum of biological activities, including potent antioxidant, anti-inflammatory, anti-cancer, and cardioprotective effects [15,37]. Its antioxidant activity primarily derives from its chemical structure, which allows it to effectively scavenge ROS, thereby preventing oxidative damage to lipids, proteins, and nucleic acids [38,39].

At the cellular and molecular levels, resveratrol exerts antioxidant effects by activating nuclear factor erythroid 2-related factor 2 (Nrf2), a transcription factor that regulates antioxidant response elements (ARE) and upregulates the expression and activity of endogenous antioxidant enzymes such as superoxide dismutase (SOD), catalase and glutathione peroxidase [40,41,42]. Furthermore, resveratrol decreases both gene and protein expression of NADPH oxidase isoforms, particularly NOX2, a major enzymatic source of reactive oxygen species (ROS) in vascular tissues under pathophysiological conditions [43,44,45].

In addition to its antioxidant properties, resveratrol also modulates endothelial function. It is known to upregulate eNOS expression and activity, enhancing NO bioavailability and improving endothelial-dependent vasodilation in animals and humans [46,47,48,49]. This protective vascular action of resveratrol is largely mediated via the activation of sirtuin-1 (SIRT1), a NAD^+^-dependent protein deacetylase [37]. SIRT1 activation by resveratrol promotes eNOS deacetylation and subsequent activation, leading to increased NO production and attenuation of oxidative-stress-induced endothelial dysfunction [50].

Resveratrol also exerts anti-inflammatory effects by inhibiting the nuclear factor-kappa B (NF-κB) signaling pathway, leading to reduced expression of pro-inflammatory cytokines and adhesion molecules such as tumor necrosis factor-alpha (TNF-α), interleukin-6 (IL-6), intercellular adhesion molecule-1 (ICAM-1), and vascular cell adhesion molecule-1 (VCAM-1) [51,52,53]. Collectively, these actions contribute to decreased vascular inflammation, reduced leukocyte infiltration, and improved expression function.

In addition, resveratrol exhibits potent anti-proliferative effects on vascular smooth muscle cells by targeting key signaling pathways involved in vascular remodeling. It suppresses mitogen-activated protein kinase (MAPK), phosphoinositide 3-kinase/protein kinase B (PI3K/Akt), and transforming growth factor-beta (TGF-β) cascades, which are implicated in pathological vascular remodeling [54,55,56].

Taken together, these antioxidant, anti-inflammatory, endothelial-protective, and anti-remodeling effects provide a mechanistic rationale for the use of resveratrol in vascular conditions characterized by oxidative stress and endothelial dysfunction. In SCD, where redox imbalance, NO deficiency, and altered vascular reactivity converge to promote priapism, these properties may hold particular therapeutic promise.

## 5. Evaluation of Resveratrol and Its Nitric Oxide–Donor Hybrid in SCD-Associated Priapism

### 5.1. Evaluation of Resveratrol Monotherapy

The therapeutic potential of resveratrol in SCD-associated priapism has been thoroughly investigated using transgenic mouse models that replicate key pathophysiological features of the human condition. These include chronic intravascular hemolysis, oxidative stress, and dysregulation of NO signaling within the corpus cavernosum. In a recent study, male SCD mice (Townes model) received oral resveratrol at a dose of 100 mg/kg/day for 14 consecutive days, and the effects were evaluated through both functional and molecular endpoints [19].

Ex vivo experiments using isolated corpus cavernosum strips mounted in organ bath chambers revealed that vehicle-treated SCD mice displayed enhanced relaxation responses to acetylcholine, sodium nitroprusside, and electrical field stimulation. These findings were consistent with the exaggerated cavernosal relaxation characteristic of the priapism phenotype [9,10,11,12]. Resveratrol treatment significantly attenuated these responses, indicating restoration of regulatory control over cavernosal smooth muscle tone. In wild-type controls, resveratrol did not alter cavernosal reactivity, supporting a disease-specific pharmacological effect rather than generalized vascular inhibition [19].

**Figure 3:**
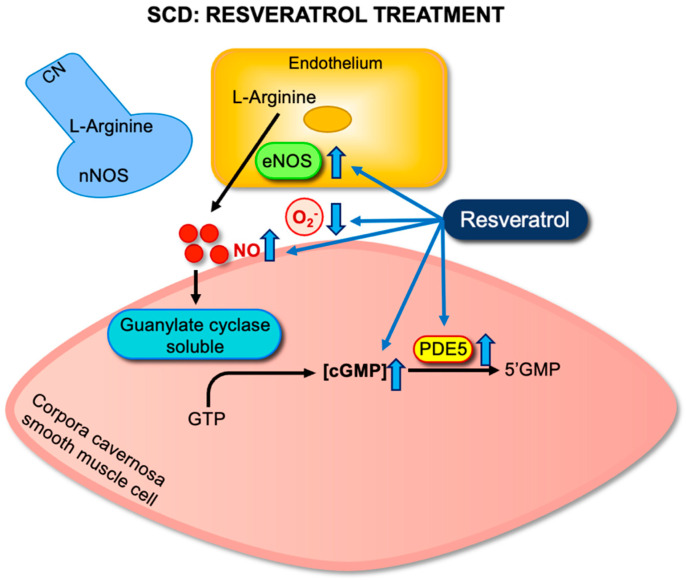
Effects of resveratrol on NO–cGMP–PDE5 signaling in sickle cell disease (SCD). Resveratrol enhances endothelial nitric oxide synthase (eNOS) expression and reduces oxidative stress, thereby increasing NO bioavailability. Improved NO signaling promotes soluble guanylate cyclase (sGC) activation and cGMP synthesis in cavernosal smooth muscle cells. Additionally, resveratrol upregulates phosphodiesterase type 5 (PDE5), restoring cGMP degradation. Together, these effects rebalance the NO–cGMP–PDE5 pathway and normalize cavernosal smooth muscle tone, potentially preventing priapism in SCD.

Mechanistic analysis showed that resveratrol reestablished important elements of the NO–cGMP–PDE5 pathway. Untreated SCD mice exhibited downregulation of eNOS and PDE5, along with reduced tissue cGMP levels. These molecular alterations reflect a dysfunctional signaling environment that predisposes to unregulated smooth muscle relaxation. Resveratrol treatment normalized eNOS and PDE5 gene expression and led to a significant increase in cavernosal cGMP content. These changes indicate improved NO production and enhanced downstream signaling efficacy [19] (Figure 3).

In parallel, resveratrol effectively reduced oxidative stress within penile tissue. Treatment led to a marked decrease in the expression of NOX2, a catalytic subunit of NADPH oxidase, which is a primary enzymatic source of ROS in this context. Furthermore, levels of oxidative stress markers, including 4-hydroxynonenal and 3-NT, were significantly decreased following resveratrol administration. These findings indicate that resveratrol enhances NO bioavailability by stimulating its synthesis and preventing its oxidative inactivation [19].

It is important to note that resveratrol treatment did not significantly modify systemic hematological parameters such as hemoglobin concentration, hematocrit, or red blood cell counts [19]. This observation reinforces the tissue-selective effect of resveratrol and suggests that its vascular benefits are achieved without altering the underlying hematological status of the SCD model, an important consideration for translational applications.

Overall, these preclinical findings demonstrate that resveratrol reverses key functional and molecular abnormalities associated with priapism in SCD. By restoring NO signaling, reducing oxidative stress, and normalizing cavernosal responsiveness, resveratrol represents a promising targeted pharmacological strategy for this debilitating complication. Clinical studies are now warranted to define its therapeutic potential and establish its efficacy in patients with SCD-related priapism.

### 5.2. Evaluation of Resveratrol–NO Donor Hybrids (RVT-FxMe)

To expand the therapeutic potential of resveratrol and enhance its pharmacodynamic properties, hybrid compounds incorporating NO donor functionalities have been developed. One such compound, I-4-(4-(4-methoxystyryl) phenoxy)-3-methyl-1,2,5-oxadiazole 2-N-oxide (RVT-FxMe or compound **10a**), was synthesized by chemically linking a NO-donating moiety to resveratrol, with the rationale of simultaneously targeting oxidative stress and NO deficiency, which are two central mechanisms in the pathophysiology of priapism in SCD [20,21]. This dual-action design integrates NO donation with the intrinsic antioxidant profile of resveratrol, thereby addressing the two core mechanisms driving priapism in SCD.

RVT-FxMe was evaluated in two murine models that exhibit the priapism phenotype: transgenic SCD mice and eNOS-deficient (eNOS^−/−^) mice [21]. Both models display exaggerated cavernosal relaxation with downregulated PDE5 protein expression. However, while the eNOS^−/−^ model represents a non-hemolytic state characterized by isolated NO deficiency, the SCD model involves a multifactorial pathophysiology, including hemolysis, oxidative stress, NO inactivation, and endothelial dysfunction [21,27]. These contrasting mechanisms provide a relevant framework for evaluating the therapeutic performance of RVT-FxMe under distinct pathophysiological conditions.

Mice were treated orally with RVT-FxMe (25 mg/kg/day) for 14 days. In eNOS^−^/^−^ mice, RVT-FxMe significantly attenuated the exaggerated cavernosal relaxation responses to SNP and EFS. These findings suggest that exogenous NO delivery via the hybrid compound is sufficient to restore downstream signaling in a model characterized by impaired NO synthesis. The effectiveness of RVT-FxMe in this context demonstrates its capacity to normalize sGC–cGMP–PDE5 signaling.

In contrast, RVT-FxMe failed to reverse the exaggerated relaxation responses in SCD mice [21]. Despite its structural similarity to resveratrol and its NO-releasing capacity, the hybrid compound did not improve functional or molecular parameters associated with priapism in this hemolytic model. This discrepancy is likely due to the high concentrations of circulating cell-free hemoglobin in SCD, which bind and inactivate NO before it reaches its smooth muscle targets. Consequently, the NO released by RVT-FxMe is rapidly scavenged, limiting its ability to restore cGMP signaling and regulate cavernosal tone.

An additional factor to consider is the difference in dosing between the two compounds. Whereas resveratrol was administered at 100 mg/kg/day, RVT-FxMe was tested at a quarter of that dose (25 mg/kg/day). Although this lower dose was chosen to reflect the presumed increased potency of the hybrid, it may have been insufficient to overcome the biochemical barriers presented by the SCD microenvironment. Dose-escalation studies are required to determine whether higher concentrations of RVT-FxMe can achieve therapeutic effects comparable to resveratrol monotherapy.

Beyond its effects on penile vascular function, RVT-FxMe has demonstrated additional pharmacological advantages compared to native resveratrol. Originally developed as part of a series of RVT derivatives (10a–i) aimed at treating SCD symptoms more broadly, RVT-FxMe was identified as the most potent in several biological assays. Unlike resveratrol, RVT-FxMe was capable of releasing NO, with NO release levels reaching up to 26.3%. In a murine model of acetic acid-induced visceral pain, RVT-FxMe produced a significant antinociceptive effect, offering up to 37.3% protection, the highest among the series. In vitro studies using LPS-stimulated macrophages showed that RVT-FxMe reduced TNF-α levels by 41.1–64.3% across tested concentrations (3.13–12.5 μM), a more robust anti-inflammatory effect than that observed with resveratrol. Moreover, both RVT-FxMe and resveratrol doubled gamma-globin (γG+γA) chain production in CD34^+^ hematopoietic cells compared to vehicle, indicating potential utility in modifying the hematological profile of SCD. RVT-FxMe did not exhibit mutagenicity or membrane-disruptive activity in safety assays. These findings suggest that RVT-FxMe may offer systemic therapeutic benefits through combined anti-inflammatory, analgesic, and gamma-globin-inducing properties, supporting its continued exploration as a multifunctional therapeutic candidate for SCD.

## 6. Potential Therapeutic Role of Resveratrol Combined with Current Treatments

The therapeutic potential of resveratrol in managing priapism associated with SCD could be significantly enhanced by combining it with current treatment options. Currently approved therapies for SCD include hydroxyurea, L-glutamine, and crizanlizumab, each targeting different aspects of the disease pathophysiology. However, these treatments primarily focus on reducing hemolysis, inflammation, and vaso-occlusive episodes, with limited direct impact on the specific oxidative and nitrosative stress mechanisms underlying priapism.

Preclinical findings have demonstrated the limited ability of hydroxyurea to directly correct cavernosal smooth muscle dysregulation in SCD. In a study using transgenic SCD and eNOS-deficient mice, hydroxyurea administered intraperitoneally for three weeks did not reverse the priapism phenotype stress [27]. These results indicate that hydroxyurea does not restore NO–cGMP signaling at the tissue level, likely due to persistent NO scavenging by free hemoglobin and ongoing oxidative stress [27]. By contrast, a clinical trial demonstrated a significant reduction in priapism frequency after a median of 10 months of hydroxyurea therapy [57]. This discrepancy likely reflects the systemic hematological benefits of long-term therapy in humans, including reduced hemolysis and lower circulating levels of cell-free hemoglobin, which were not reproduced in the short-term animal protocol. These upstream effects may secondarily improve endothelial function and smooth muscle homeostasis in the penis.

Together, these findings suggest that while hydroxyurea may not directly normalize molecular signaling pathways involved in priapism at the penile level, its systemic effects can indirectly reduce the occurrence and severity of priapism episodes. This reinforces the potential value of adjunctive agents like resveratrol that act locally to restore redox balance and NO bioactivity within the corpus cavernosum. Combining systemic hematologic correction with local vascular modulation may offer additive or synergistic benefits.

L-glutamine is the first FDA-approved antioxidant therapy for SCD, shown to reduce the frequency of vaso-occlusive crises by improving the redox potential of sickled erythrocytes through increased NAD synthesis [58]. Although its systemic antioxidant effects are clinically relevant, its impact on localized vascular complications, such as priapism, remains unexplored. In contrast, resveratrol exerts both systemic and tissue-level antioxidant effects, including direct modulation of endothelial NO bioavailability. These complementary mechanisms suggest that resveratrol could enhance the benefits of L-glutamine, and future studies should evaluate their combined potential to provide broader vascular protection in SCD.

Despite prior positive findings from the SUSTAIN trial, the recent STAND phase 3 study failed to demonstrate significant efficacy of crizanlizumab over placebo in reducing the frequency of vaso-occlusive crises, although its safety profile was confirmed [59,60]. Following these disappointing results, crizanlizumab was withdrawn from the European market in 2023 and subsequently from the Brazilian market. These outcomes clearly demonstrate the limitations of anti-adhesive monotherapy and reinforce the urgent need for alternative or combinatorial strategies targeting multiple pathophysiological mechanisms.

Collectively, integrating resveratrol with established SCD therapies offers a promising strategy, particularly for complications such as priapism that are driven by local oxidative and endothelial dysfunction. By complementing the systemic effects of current treatments, resveratrol may help normalize tissue-specific redox signaling and restore NO bioavailability in the penis. Translational studies combining pharmacokinetic, molecular, and functional endpoints are needed to confirm these potential synergistic effects and support future clinical applications.

## 7. Clinical Evidence and Translational Challenges

Although most evidence supporting the therapeutic potential of resveratrol comes from preclinical models, an increasing number of clinical studies have examined its effects on vascular health [61]. These trials consistently report that resveratrol is well tolerated at doses up to 1 g/day, with only mild gastrointestinal symptoms, such as flatulence, nausea, or diarrhea, observed at higher doses (2.5–5 g/day) [61,62,63,64]. This favorable safety profile supports its continued investigation as an adjunctive therapeutic agent for vascular dysfunction.

The clinical efficacy of resveratrol remains inconsistent. Randomized controlled trials have reported variable effects on endothelial and vascular biomarkers, often without a clear dose–response relationship [61]. This variability may reflect differences in study design, participant characteristics, treatment duration, formulation, and the absence of standardized outcome measures. Moreover, the clinical effects reported are frequently modest when compared to those observed in animal models [61].

A central barrier to clinical translation is the poor pharmacokinetic profile of resveratrol. Although trans-resveratrol demonstrates high oral absorption, its systemic bioavailability is extremely limited due to rapid and extensive first-pass metabolism. Sulfate and glucuronide conjugation of phenolic hydroxyl groups are the primary metabolic routes, with intestinal and hepatic sulfation representing a critical rate-limiting step [65]. The half-life of trans-resveratrol ranges from 1 to 3 h after a single dose and extends to 2 to 5 h following repeated administration [66]. Circadian variation has also been observed, with higher bioavailability occurring after morning intake [66]. Despite low plasma levels of the parent compound, the biological activity of its conjugated metabolites remains poorly characterized and should be addressed in future studies.

Beyond systemic pharmacokinetics, tissue distribution and accumulation are important to therapeutic efficacy but remain underexplored. Plasma concentrations may not adequately reflect local bioactivity at target sites. Preclinical evidence has shown that resveratrol metabolites can accumulate in myocardial tissue in a dose- and time-dependent manner, with levels correlating to functional hemodynamic outcomes in diabetic rats [67]. Whether similar tissue-level bioavailability is achieved in the corpus cavernosum remains unknown. Quantifying local concentrations and pharmacodynamic responses at the site of action is essential for understanding therapeutic mechanisms and optimizing dosage strategies.

To address these limitations, multiple strategies have been proposed, including nanoformulations, liposomal carriers, and structural modifications such as hybridization with NO donor groups. These approaches aim to enhance oral bioavailability, improve tissue penetration, and reduce metabolic degradation. However, their effectiveness in improving site-specific drug delivery remains to be validated.

## 8. Conclusions

Resveratrol represents a promising therapeutic approach for oxidative-stress-mediated priapism in SCD, acting through the restoration of NO bioavailability, reduction in oxidative stress, and normalization of cavernosal smooth muscle tone. Although NO-donor hybrids have not yet demonstrated efficacy under hemolytic conditions, their development demonstrates the need for optimized delivery strategies. The favorable safety profile of resveratrol supports its continued investigation as an adjunct to existing therapies for this debilitating complication.

## References

[1] Kato G.J., Piel F.B., Reid C.D., Gaston M.H., Ohene-Frempong K., Krishnamurti L., Smith W.R., Panepinto J.A., Weatherall D.J., Costa F.F. (2018). Sickle Cell Disease. Nat. Rev. Dis. Primer.

[2] Arduini G.A.O., Trovó de Marqui A.B. (2018). Prevalence and Characteristics of Priapism in Sickle Cell Disease. Hemoglobin.

[3] Bivalacqua T.J., Allen B.K., Brock G.B., Broderick G.A., Chou R., Kohler T.S., Mulhall J.P., Oristaglio J., Rahimi L.L., Rogers Z.R. (2022). The Diagnosis and Management of Recurrent Ischemic Priapism, Priapism in Sickle Cell Patients, and Non-Ischemic Priapism: An AUA/SMSNA Guideline. J. Urol..

[4] Silveira T.H.R., Pereira D.A., Calmasini F.B., Costa F.F., Burnett A.L., Silva F.H. (2024). Sympathetic Hypoactivity Leads to Hypocontractility of the Corpus Cavernosum in Sickle Cell Mice: A Mechanism Contributing to Priapism. Int. J. Impot. Res..

[5] Musicki B., Burnett A.L. (2020). Mechanisms Underlying Priapism in Sickle Cell Disease: Targeting and Key Innovations on the Preclinical Landscape. Expert Opin. Ther. Targets.

[6] Pereira D.A., Calmasini F.B., Costa F.F., Burnett A.L., Silva F.H. (2024). Nitric Oxide Resistance in Priapism Associated with Sickle Cell Disease: Mechanisms, Therapeutic Challenges, and Future Directions. J. Pharmacol. Exp. Ther..

[7] Musicki B., Liu T., Sezen S.F., Burnett A.L. (2012). Targeting NADPH Oxidase Decreases Oxidative Stress in the Transgenic Sickle Cell Mouse Penis. J. Sex. Med..

[8] Bivalacqua T.J., Musicki B., Hsu L.L., Berkowitz D.E., Champion H.C., Burnett A.L. (2013). Sildenafil Citrate-Restored eNOS and PDE5 Regulation in Sickle Cell Mouse Penis Prevents Priapism via Control of Oxidative/Nitrosative Stress. PLoS ONE.

[9] Silva F.H., Claudino M.A., Calmasini F.B., Alexandre E.C., Franco-Penteado C., Burnett A.L., Antunes E., Costa F.F. (2016). Sympathetic Hyperactivity, Increased Tyrosine Hydroxylase and Exaggerated Corpus Cavernosum Relaxations Associated with Oxidative Stress Plays a Major Role in the Penis Dysfunction in Townes Sickle Cell Mouse. PLoS ONE.

[10] Pereira P.d.S., Pereira D.A., Calmasini F.B., Reis L.O., Brinkman N., Burnett A.L., Costa F.F., Silva F.H. (2022). Haptoglobin Treatment Contributes to Regulating Nitric Oxide Signal and Reduces Oxidative Stress in the Penis: A Preventive Treatment for Priapism in Sickle Cell Disease. Front. Physiol..

[11] Silva F.H., Karakus S., Musicki B., Matsui H., Bivalacqua T.J., Dos Santos J.L., Costa F.F., Burnett A.L. (2016). Beneficial Effect of the Nitric Oxide Donor Compound 3-(1,3-Dioxoisoindolin-2-Yl)Benzyl Nitrate on Dysregulated Phosphodiesterase 5, NADPH Oxidase, and Nitrosative Stress in the Sickle Cell Mouse Penis: Implication for Priapism Treatment. J. Pharmacol. Exp. Ther..

[12] Musicki B., Karakus S., Akakpo W., Silva F.H., Liu J., Chen H., Zirkin B.R., Burnett A.L. (2018). Testosterone Replacement in Transgenic Sickle Cell Mice Controls Priapic Activity and Upregulates PDE5 Expression and eNOS Activity in the Penis. Andrology.

[13] Musicki B., Karakus S., La Favor J.D., Chen H., Silva F.H., Sturny M., Zirkin B.R., Burnett A.L. (2020). TSPO Ligand FGIN-1-27 Controls Priapism in Sickle Cell Mice via Endogenous Testosterone Production. J. Cell. Physiol..

[14] Fukuhara S., Tsujimura A., Okuda H., Yamamoto K., Takao T., Miyagawa Y., Nonomura N., Okuyama A. (2011). Vardenafil and Resveratrol Synergistically Enhance the Nitric Oxide/Cyclic Guanosine Monophosphate Pathway in Corpus Cavernosal Smooth Muscle Cells and Its Therapeutic Potential for Erectile Dysfunction in the Streptozotocin-Induced Diabetic Rat: Preliminary Findings. J. Sex. Med..

[15] Bonnefont-Rousselot D. (2016). Resveratrol and Cardiovascular Diseases. Nutrients.

[16] Sener T.E., Tavukcu H.H., Atasoy B.M., Cevik O., Kaya O.T., Cetinel S., Dagli Degerli A., Tinay I., Simsek F., Akbal C. (2018). Resveratrol Treatment May Preserve the Erectile Function after Radiotherapy by Restoring Antioxidant Defence Mechanisms, SIRT1 and NOS Protein Expressions. Int. J. Impot. Res..

[17] Yazir Y., Şahin T.D., Furat Rençber S., Gacar G., Halbutoğulları Z.S., Utkan T., Aricioglu F. (2018). Restorative Effect of Resveratrol on Expression of Endothelial and Neuronal Nitric Oxide Synthase in Cavernous Tissues of Chronic Unpredictable Mild Stress-Exposed Rats: An Impact of Inflammation. Int. J. Impot. Res..

[18] Yu W., Wang J., Dai Y.-T., Wang B., Xu Y., Gao Q.-Q., Xu Z.-P. (2022). Modulation of SIRT1 Expression Improves Erectile Function in Aged Rats. Asian J. Androl..

[19] Splendore C.O., Silveira T.H.R., Pereira D.A., Bossarino B.P., de Oliveira M.G., Calmasini F.B., Burnett A.L., Costa F.F., Silva F.H. (2025). Resveratrol Attenuates the Priapism Phenotype in Sickle Cell Mice by Restoring NO-cGMP-PDE5 Signaling and Reducing NADPH Oxidase 2 Expression. Front. Pharmacol..

[20] Bosquesi P.L., Melchior A.C.B., Pavan A.R., Lanaro C., de Souza C.M., Rusinova R., Chelucci R.C., Barbieri K.P., Fernandes G.F.d.S., Carlos I.Z. (2020). Synthesis and Evaluation of Resveratrol Derivatives as Fetal Hemoglobin Inducers. Bioorg. Chem..

[21] Pinheiro A.K., Pereira D.A., Dos Santos J.L., Calmasini F.B., Alexandre E.C., Reis L.O., Burnett A.L., Costa F.F., Silva F.H. (2022). Resveratrol-Nitric Oxide Donor Hybrid Effect on Priapism in Sickle Cell and Nitric Oxide-Deficient Mouse. PLoS ONE.

[22] MacDonald S.M., Burnett A.L. (2021). Physiology of Erection and Pathophysiology of Erectile Dysfunction. Urol. Clin. N. Am..

[23] Andersson K.-E. (2011). Mechanisms of Penile Erection and Basis for Pharmacological Treatment of Erectile Dysfunction. Pharmacol. Rev..

[24] Champion H.C., Bivalacqua T.J., Takimoto E., Kass D.A., Burnett A.L. (2005). Phosphodiesterase-5A Dysregulation in Penile Erectile Tissue Is a Mechanism of Priapism. Proc. Natl. Acad. Sci. USA.

[25] Lagoda G., Sezen S.F., Cabrini M.R., Musicki B., Burnett A.L. (2013). Molecular Analysis of Erection Regulatory Factors in Sickle Cell Disease-Associated Priapism in Human Penis. J. Urol..

[26] Lin C.-S., Chow S., Lau A., Tu R., Lue T.F. (2002). Human PDE5A Gene Encodes Three PDE5 Isoforms from Two Alternate Promoters. Int. J. Impot. Res..

[27] Pereira D.A., Pereira D.A., Pereira P.d.S., Silveira T.H.R., Calmasini F.B., Reis L.O., Costa F.F., Silva F.H. (2023). Hydroxyurea Does Not Reverse Functional Alterations of the Nitric Oxide-cGMP Pathway Associated with Priapism Phenotype in Corpus Cavernosum from Sickle Cell Mouse. PLoS ONE.

[28] Lagoda G., Sezen S.F., Hurt K.J., Cabrini M.R., Mohanty D.K., Burnett A.L. (2014). Sustained Nitric Oxide (NO)-Releasing Compound Reverses Dysregulated NO Signal Transduction in Priapism. FASEB J..

[29] Reiter C.D., Wang X., Tanus-Santos J.E., Hogg N., Cannon R.O., Schechter A.N., Gladwin M.T. (2002). Cell-Free Hemoglobin Limits Nitric Oxide Bioavailability in Sickle-Cell Disease. Nat. Med..

[30] Iacopucci A.P.M., da Silva Pereira P., Pereira D.A., Calmasini F.B., Pittalà V., Reis L.O., Burnett A.L., Costa F.F., Silva F.H. (2022). Intravascular Hemolysis Leads to Exaggerated Corpus Cavernosum Relaxation: Implication for Priapism in Sickle Cell Disease. FASEB J. Off. Publ. Fed. Am. Soc. Exp. Biol..

[31] Schmidt H.M., Kelley E.E., Straub A.C. (2019). The Impact of Xanthine Oxidase (XO) on Hemolytic Diseases. Redox Biol..

[32] Pacher P., Beckman J.S., Liaudet L. (2007). Nitric Oxide and Peroxynitrite in Health and Disease. Physiol. Rev..

[33] Musicki B., Bivalacqua T.J., Champion H.C., Burnett A.L. (2014). Sildenafil Promotes eNOS Activation and Inhibits NADPH Oxidase in the Transgenic Sickle Cell Mouse Penis. J. Sex. Med..

[34] Fu S., Tar M.T., Melman A., Davies K.P. (2014). Opiorphin Is a Master Regulator of the Hypoxic Response in Corporal Smooth Muscle Cells. FASEB J. Off. Publ. Fed. Am. Soc. Exp. Biol..

[35] Ning C., Wen J., Zhang Y., Dai Y., Wang W., Zhang W., Qi L., Grenz A., Eltzschig H.K., Blackburn M.R. (2014). Excess Adenosine A2B Receptor Signaling Contributes to Priapism through HIF-1α Mediated Reduction of PDE5 Gene Expression. FASEB J. Off. Publ. Fed. Am. Soc. Exp. Biol..

[36] Bivalacqua T.J., Ross A.E., Strong T.D., Gebska M.A., Musicki B., Champion H.C., Burnett A.L. (2010). Attenuated RhoA/Rho-Kinase Signaling in Penis of Transgenic Sickle Cell Mice. Urology.

[37] Xia N., Daiber A., Förstermann U., Li H. (2017). Antioxidant Effects of Resveratrol in the Cardiovascular System. Br. J. Pharmacol..

[38] Leonard S.S., Xia C., Jiang B.-H., Stinefelt B., Klandorf H., Harris G.K., Shi X. (2003). Resveratrol Scavenges Reactive Oxygen Species and Effects Radical-Induced Cellular Responses. Biochem. Biophys. Res. Commun..

[39] Jomova K., Raptova R., Alomar S.Y., Alwasel S.H., Nepovimova E., Kuca K., Valko M. (2023). Reactive Oxygen Species, Toxicity, Oxidative Stress, and Antioxidants: Chronic Diseases and Aging. Arch. Toxicol..

[40] Chen C.-Y., Jang J.-H., Li M.-H., Surh Y.-J. (2005). Resveratrol Upregulates Heme Oxygenase-1 Expression via Activation of NF-E2-Related Factor 2 in PC12 Cells. Biochem. Biophys. Res. Commun..

[41] Rubiolo J.A., Mithieux G., Vega F.V. (2008). Resveratrol Protects Primary Rat Hepatocytes against Oxidative Stress Damage: Activation of the Nrf2 Transcription Factor and Augmented Activities of Antioxidant Enzymes. Eur. J. Pharmacol..

[42] Javkhedkar A.A., Quiroz Y., Rodriguez-Iturbe B., Vaziri N.D., Lokhandwala M.F., Banday A.A. (2015). Resveratrol Restored Nrf2 Function, Reduced Renal Inflammation, and Mitigated Hypertension in Spontaneously Hypertensive Rats. Am. J. Physiol. Regul. Integr. Comp. Physiol..

[43] Guo S., Yao Q., Ke Z., Chen H., Wu J., Liu C. (2015). Resveratrol Attenuates High Glucose-Induced Oxidative Stress and Cardiomyocyte Apoptosis through AMPK. Mol. Cell. Endocrinol..

[44] Yao Y., Li J., Niu Y., Yu J.-Q., Yan L., Miao Z.-H., Zhao X.-X., Li Y.-J., Yao W.-X., Zheng P. (2015). Resveratrol Inhibits Oligomeric Aβ-Induced Microglial Activation via NADPH Oxidase. Mol. Med. Rep..

[45] Huang X., Zhao W., Hu D., Han X., Wang H., Yang J., Xu Y., Li Y., Yao W., Chen C. (2017). Resveratrol Efficiently Improves Pulmonary Function via Stabilizing Mast Cells in a Rat Intestinal Injury Model. Life Sci..

[46] Csiszar A., Labinskyy N., Podlutsky A., Kaminski P.M., Wolin M.S., Zhang C., Mukhopadhyay P., Pacher P., Hu F., de Cabo R. (2008). Vasoprotective Effects of Resveratrol and SIRT1: Attenuation of Cigarette Smoke-Induced Oxidative Stress and Proinflammatory Phenotypic Alterations. Am. J. Physiol. Heart Circ. Physiol..

[47] Carrizzo A., Puca A., Damato A., Marino M., Franco E., Pompeo F., Traficante A., Civitillo F., Santini L., Trimarco V. (2013). Resveratrol Improves Vascular Function in Patients with Hypertension and Dyslipidemia by Modulating NO Metabolism. Hypertension.

[48] Cheang W.S., Wong W.T., Wang L., Cheng C.K., Lau C.W., Ma R.C.W., Xu A., Wang N., Huang Y., Tian X.Y. (2019). Resveratrol Ameliorates Endothelial Dysfunction in Diabetic and Obese Mice through Sirtuin 1 and Peroxisome Proliferator-Activated Receptor δ. Pharmacol. Res..

[49] Li J., Feng Z., Lu B., Fang X., Huang D., Wang B. (2023). Resveratrol Alleviates High Glucose-Induced Oxidative Stress and Apoptosis in Rat Cardiac Microvascular Endothelial Cell through AMPK/Sirt1 Activation. Biochem. Biophys. Rep..

[50] Arunachalam G., Yao H., Sundar I.K., Caito S., Rahman I. (2010). SIRT1 Regulates Oxidant- and Cigarette Smoke-Induced eNOS Acetylation in Endothelial Cells: Role of Resveratrol. Biochem. Biophys. Res. Commun..

[51] Pan W., Yu H., Huang S., Zhu P. (2016). Resveratrol Protects against TNF-α-Induced Injury in Human Umbilical Endothelial Cells through Promoting Sirtuin-1-Induced Repression of NF-KB and P38 MAPK. PLoS ONE.

[52] Liu C.-W., Sung H.-C., Lin S.-R., Wu C.-W., Lee C.-W., Lee I.-T., Yang Y.-F., Yu I.-S., Lin S.-W., Chiang M.-H. (2017). Resveratrol Attenuates ICAM-1 Expression and Monocyte Adhesiveness to TNF-α-Treated Endothelial Cells: Evidence for an Anti-Inflammatory Cascade Mediated by the miR-221/222/AMPK/P38/NF-κB Pathway. Sci. Rep..

[53] Nallasamy P., Kang Z.Y., Sun X., Anandh Babu P.V., Liu D., Jia Z. (2021). Natural Compound Resveratrol Attenuates TNF-Alpha-Induced Vascular Dysfunction in Mice and Human Endothelial Cells: The Involvement of the NF-κB Signaling Pathway. Int. J. Mol. Sci..

[54] Chen B., Xue J., Meng X., Slutzky J.L., Calvert A.E., Chicoine L.G. (2014). Resveratrol Prevents Hypoxia-Induced Arginase II Expression and Proliferation of Human Pulmonary Artery Smooth Muscle Cells via Akt-Dependent Signaling. Am. J. Physiol. Lung Cell. Mol. Physiol..

[55] Almajdoob S., Hossain E., Anand-Srivastava M.B. (2018). Resveratrol Attenuates Hyperproliferation of Vascular Smooth Muscle Cells from Spontaneously Hypertensive Rats: Role of ROS and ROS-Mediated Cell Signaling. Vascul. Pharmacol..

[56] Fan J., Wei S., Zhang X., Chen L., Zhang X., Jiang Y., Sheng M., Chen Y. (2023). Resveratrol Inhibits TGF-Β1-Induced Fibrotic Effects in Human Pterygium Fibroblasts. Environ. Health Prev. Med..

[57] Idris I.M., Yusuf A.A., Ismail I.I., Borodo A.M., Hikima M.S., Kana S.A., Aliyu T., Musangedu K., Jibrilla A.U., Aji S.A. (2025). A Controlled Trial for Preventing Priapism in Sickle Cell Anemia: Hydroxyurea plus Placebo vs Hydroxyurea plus Tadalafil. Blood.

[58] Niihara Y., Miller S.T., Kanter J., Lanzkron S., Smith W.R., Hsu L.L., Gordeuk V.R., Viswanathan K., Sarnaik S., Osunkwo I. (2018). A Phase 3 Trial of L-Glutamine in Sickle Cell Disease. N. Engl. J. Med..

[59] Ataga K.I., Kutlar A., Kanter J., Liles D., Cancado R., Friedrisch J., Guthrie T.H., Knight-Madden J., Alvarez O.A., Gordeuk V.R. (2017). Crizanlizumab for the Prevention of Pain Crises in Sickle Cell Disease. N. Engl. J. Med..

[60] Abboud M.R., Cançado R.D., Montalembert M.D., Smith W.R., Rimawi H., Voskaridou E., Güvenç B., Ataga K.I., Keefe D., Grosch K. (2025). Crizanlizumab with or without Hydroxyurea in Patients with Sickle Cell Disease (STAND): Primary Analyses from a Placebo-Controlled, Randomised, Double-Blind, Phase 3 Trial. Lancet Haematol..

[61] Godos J., Romano G.L., Gozzo L., Laudani S., Paladino N., Dominguez Azpíroz I., Martínez López N.M., Giampieri F., Quiles J.L., Battino M. (2024). Resveratrol and Vascular Health: Evidence from Clinical Studies and Mechanisms of Actions Related to Its Metabolites Produced by Gut Microbiota. Front. Pharmacol..

[62] Boocock D.J., Faust G.E.S., Patel K.R., Schinas A.M., Brown V.A., Ducharme M.P., Booth T.D., Crowell J.A., Perloff M., Gescher A.J. (2007). Phase I Dose Escalation Pharmacokinetic Study in Healthy Volunteers of Resveratrol, a Potential Cancer Chemopreventive Agent. Cancer Epidemiol. Biomark. Prev..

[63] la Porte C., Voduc N., Zhang G., Seguin I., Tardiff D., Singhal N., Cameron D.W. (2010). Steady-State Pharmacokinetics and Tolerability of Trans-Resveratrol 2000 Mg Twice Daily with Food, Quercetin and Alcohol (Ethanol) in Healthy Human Subjects. Clin. Pharmacokinet..

[64] Chachay V.S., Macdonald G.A., Martin J.H., Whitehead J.P., O’Moore-Sullivan T.M., Lee P., Franklin M., Klein K., Taylor P.J., Ferguson M. (2014). Resveratrol Does Not Benefit Patients with Nonalcoholic Fatty Liver Disease. Clin. Gastroenterol. Hepatol. Off. Clin. Pract. J. Am. Gastroenterol. Assoc..

[65] Walle T., Hsieh F., DeLegge M.H., Oatis J.E., Walle U.K. (2004). High Absorption but Very Low Bioavailability of Oral Resveratrol in Humans. Drug Metab. Dispos. Biol. Fate Chem..

[66] Almeida L., Vaz-da-Silva M., Falcão A., Soares E., Costa R., Loureiro A.I., Fernandes-Lopes C., Rocha J.-F., Nunes T., Wright L. (2009). Pharmacokinetic and Safety Profile of Trans-Resveratrol in a Rising Multiple-Dose Study in Healthy Volunteers. Mol. Nutr. Food Res..

[67] Bresciani L., Calani L., Bocchi L., Delucchi F., Savi M., Ray S., Brighenti F., Stilli D., Del Rio D. (2014). Bioaccumulation of Resveratrol Metabolites in Myocardial Tissue Is Dose-Time Dependent and Related to Cardiac Hemodynamics in Diabetic Rats. Nutr. Metab. Cardiovasc. Dis. NMCD.

